# Aspergillus mural endocarditis presenting with multiple cerebral abscesses

**DOI:** 10.1186/s13019-018-0796-4

**Published:** 2018-10-16

**Authors:** Andrew A. Pavlina, Jared W. Peacock, Saad A. Ranginwala, Peter M. Pavlina, Joshua Ahier, Courtney R. Hanak

**Affiliations:** 1 University of Cincinnati Department of Radiology, 234 Goodman Street, Cincinnati, OH 45267 USA; 2 Eastern Virginia Medical School, 825 Fairfax Ave, Norfolk, VA 23507 USA; 3 Kettering Memorial Hospital Division of Cardiothoracic and Vascular Surgery, 3533 Southern Blvd #5650, Kettering, OH 45419 USA; 4 The Jewish Hospital Department of Surgery, 4777 E Galbraith Rd, Cincinnati, OH 45236 USA

**Keywords:** Antibiotics, Antifungal, Antiviral agents, Embolism, Endocarditis, Infection, Thoracotomy

## Abstract

**Background:**

Fungal endocarditis is a rare and lethal cardiac infection which most commonly presents in immunocompromised patients or patients with other predisposing conditions. In a small subset of these patients, lesions present as mural masses and do not have any involvement with native valves or implanted devices. Here we present one such case which was diagnosed in the antemortem period in time to be managed with surgical resection.

**Case presentation:**

A 70 year-old female patient who presented with multiple cerebral abscesses and was found on echocardiography to have a mass along the inferior wall of the left ventricle. She underwent surgical resection which revealed an Aspergillus vegetation along the left ventricle wall without any involvement of the cardiac valves. An intraoperative photograph was obtained and is presented in this case. The patient was started on antifungal therapy and expired on day 30 of treatment.

**Conclusions:**

Fungal endocarditis is a rare yet lethal disease. It can be difficult to detect and workup should be initiated immediately if there is any clinical suspicion. This is especially true in any patient with predisposing conditions or any patient who presents with undiagnosed, culture-negative fevers or evidence of embolic foci. Once diagnosis is made, early initiation of antifungal therapy coupled with aggressive surgical debridement is required for any significant chance of survival.

## Background

Fungal endocarditis is a rare and lethal cardiac infection, with Aspergillus species accounting for 20–30% of cases [1]. Cases most commonly present in immunocompromised patients or patients with predisposing conditions, including prior cardiac surgery, underlying cardiac abnormality, implanted devices, prolonged antibiotic use, malignancy, or history of solid organ or bone marrow transplant [1, 2]. In a small subset of these patients, lesions present as mural masses and do not have any involvement with native valves or implanted devices [3]. There are only a few reports of such cases being diagnosed in the antemortem period in time to be managed with surgical resection [4–6]. We present one such case in which Aspergillus mural endocarditis was discovered in an immunocompromised patient who presented with multiple rim-enhancing brain lesions but no signs of systemic infection.

## Case presentation

A 70 year old woman with a history of B-Cell Chronic Lymphocytic Leukemia (B-CLL) presented with a several week history of progressive right upper extremity weakness and increasing confusion. She had no prior history of cardiac surgery or cardiac abnormalities. Pertinent physical exam findings included a normal cardiac exam and 3/5 strength in the right arm. She was afebrile and her white cell count was at her baseline but markedly elevated (> 170 × 10^3^/μL).

**Fig. 1:**
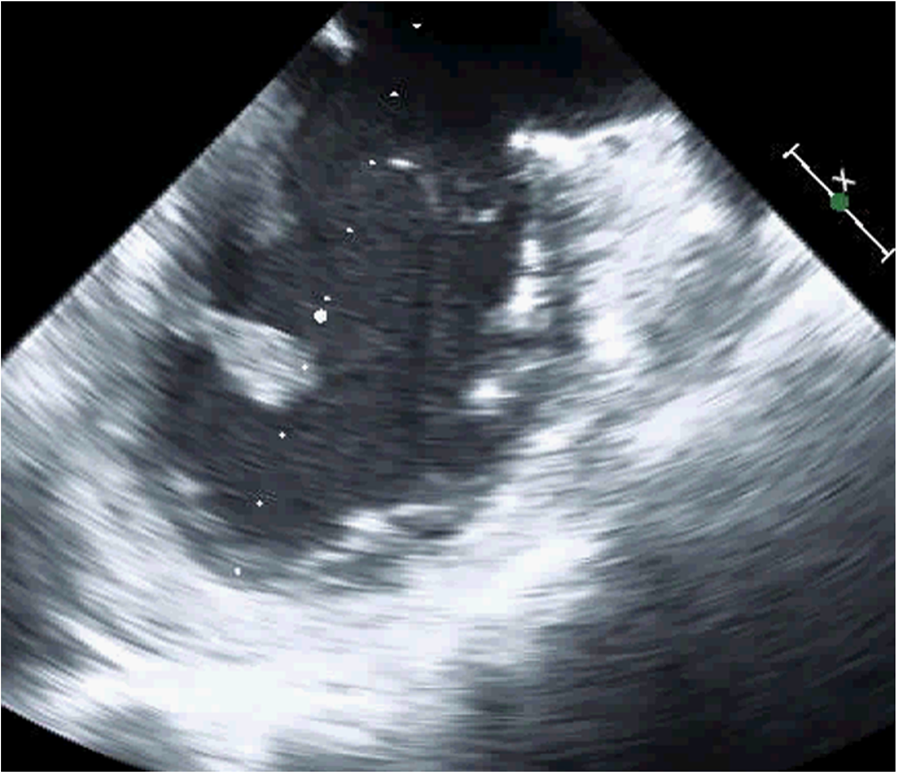
Echocardiogram demonstrating a pedunculated left ventricular vegetation

**Fig. 2:**
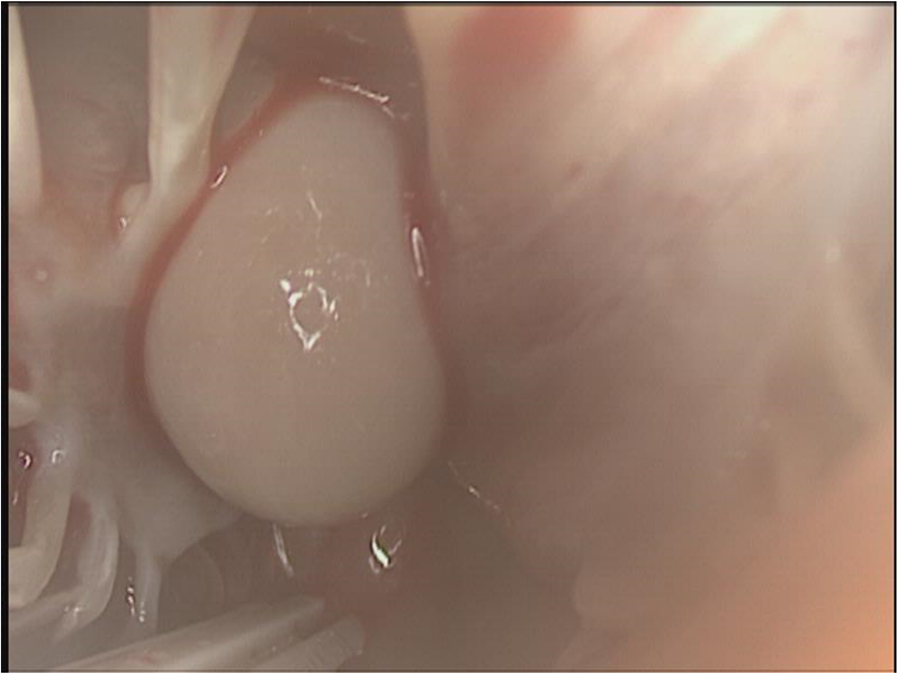
Intraoperative photograph of a smooth mass adhered to the wall of the left ventricle

**Fig. 3:**
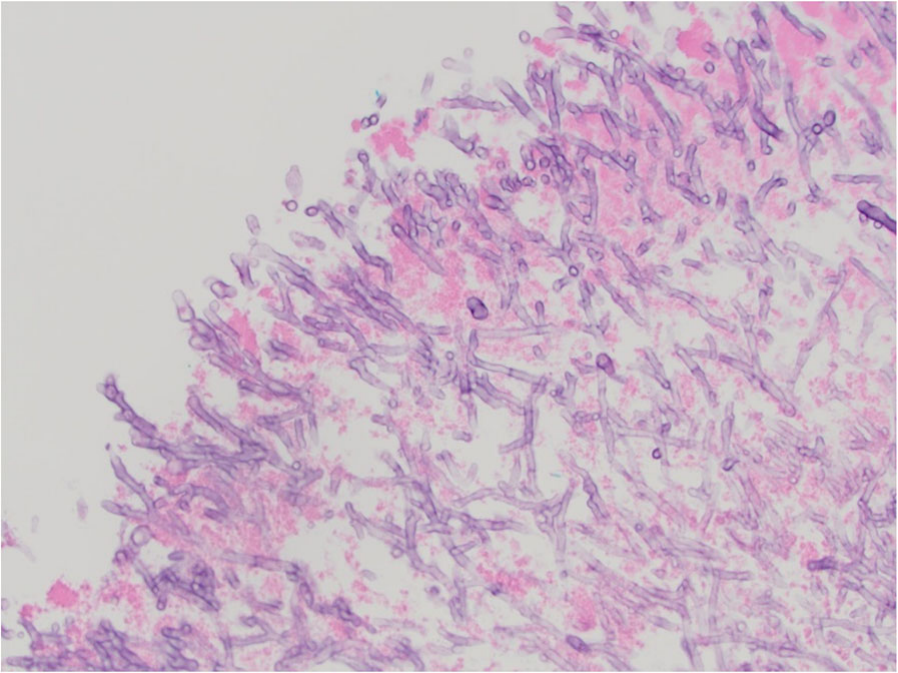
Photomicrograph of the left ventricular mass demonstrating cardiac muscle and necrotic debris with numerous fungal organisms (H&E stain, 40× magnification)

The initial workup was focused on the patient’s neurologic complaints. A brain MRI revealed multiple bilateral rim-enhancing brain lesions with surrounding vasogenic edema. The differential diagnosis included embolic or metastatic lesions, with a high suspicion for metastatic disease given the patient’s presentation and history. Workup for primary malignancy was negative, and the patient subsequently underwent a right craniotomy for biopsy. The resected lesion was found contain necrotic tissue with branching fungal hyphae suggestive of Aspergillus. Tissue cultures revealed *Aspergillus Fumigatus* species. IV Voraconazole (6 mg/kg every 12 h day 1 then 4 mg/kg every 12 h) and Micafungin (100 mg daily) were started. The Galactomannan assay was positive but all blood cultures were negative. A 2D echocardiogram was performed to evaluate for possible embolic source for the brain abscesses. This revealed a 1.3 × 1.1 cm pedunculated mass in the inferior wall of the left ventricle (Fig. 1). There was no evidence of any cardiac dysfunction and the LV ejection fraction was measured at 55%. Cardiac surgery was consulted and the patient underwent median sternotomy with cardiopulmonary bypass for removal of the left ventricular mass using a left atriotomy incision. The mitral valve was first inspected through the atriotomy incision and demonstrated no vegetations. Subsequently, a retractor was placed through the mitral valve and the left ventricular mass was easily identified. A 1 cm soft, smooth appearing mass was found adhered to the inferior septal portion of the left ventricle with surrounding inflammation(Fig. 2). It drained a small amount of purulent material which had to be suctioned. The mass was then removed and the involved cardiac muscle was debrided. There was no cardiac valve or papillary muscle involvement. Pathology and cultures again demonstrated growth of *Aspergillus Fumigatus* (Fig. 3).

The patient awakened and responded appropriately following surgery but remained in the ICU. She subsequently developed multi organ failure secondary to gram negative sepsis and expired on post-operative day 17 and day 30 of IV antifungal therapy.

## Discussion

Aspergillus species are ubiquitous in nature but rarely cause infection in immunocompetent hosts [7]. Conditions caused by Aspergillus can be categorized as noninvasive, including allergic or saprophytic (aspergilloma) forms, or invasive, in which case there is local spread or hematogenous dissemination from the respiratory tract or another source [8]. Cardiac involvement (endocarditis, pancarditis, or pericarditis) accounts for only 0.7–6% of cases of invasive aspergillosis [6, 8]. In these cases, *Aspergillus Fumigatus* the most commonly isolated pathogen [1].

Cases of aspergillus endocarditis most commonly occur in patients with predisposing conditions including prior cardiac surgery, underlying cardiac abnormality, implanted devices, prolonged antibiotic use, malignancy, immunocompromised state, or history of solid organ or bone marrow transplant [1, 2]. It is proposed that aspergillus endocarditis is caused by circulating hyphal fragments which implant on damaged or even normal myocardial tissue [3]. The majority of cases in the literature present with vegetations involving native or prosthetic valves [1, 3]. In the case presented here, there was an isolated vegetation on the left ventricular wall and no valvular involvement. It is not well-known what percentage of patients with aspergillus endocarditis have isolated mural involvement, but it has been reported to be up to 41% in autopsy reports [3]. In 1986, Mullen et al. described the first case in which aspergillus mural endocarditis was diagnosed early enough to be treated with combined medical therapy and surgical resection [5]. There have been a few similar subsequent case reports, including a recent report of aspergillus mural endocarditis in an immunocompetent patient with no known risk factors [6].

Patients with aspergillus endocarditis frequently have evidence of embolic disease on initial presentation [1]. Additionally, they may present with a new heart murmur or febrile illness with negative blood cultures [3, 6]. There should be clinical concern for fungal endocarditis in any of these cases, especially if the patient has any predisposing conditions. Workup should include transesophageal echocardiography. While aspergillus vegetations can be very large, they may be missed on transthoracic echocardiography [1, 3]. Confirming the diagnosis of aspergillus endocarditis can be very difficult. This is in large part due to the fact that fungal cultures are almost always negative with a recently estimated yield of 4% [2]. The galactomannan assay, a test for an aspergillus cell wall component which was positive in the case presented here, is more sensitive than fungal blood cultures. It still has limited diagnostic value, especially with the aspergillus fumigatus species [1, 6]. The gold standard for diagnosis of aspergillus is direct microscopic examination, often with H&E and Grocott’s methenamine silver staining, and aspergillus culture confirmation.

Prognosis in patients with aspergillus endocarditis is poor, with an estimated 96% mortality rate in patients treated with medical therapy alone [2]. Endocardial lesions require aggressive surgical debridement in order to increase survival and lower the risk for further embolic events [6–8]. First line medical therapy is Voriconazole (6 mg/kg IV every 12 h for 1 day then 4 mg/kg IV every 12 h). Additional agents including micafungin, caspofungin, and posaconazole may be used [8]. Given the risk for recurrent infections, lifelong therapy is often recommended [8].

## Conclusions

Fungal endocarditis is a rare yet lethal disease. It can be difficult to detect and workup should be initiated immediately if there is any clinical suspicion. This is especially true in any patient with predisposing conditions or any patient who presents with undiagnosed, culture-negative fevers or evidence of embolic foci. Once diagnosis is made, early initiation of antifungal therapy coupled with aggressive surgical debridement is required for any significant chance of survival.

## Abbreviations

B-CLL: B-Cell chronic lymphocytic leukemia
H + E: Hematoxylin and eosin
ICU: intensive care unit
IV: Intravenous
LV: Left ventricular
MRI: Magnetic resonance imaging

## References

[1] Kalokhe AS, Rouphael N, El Chami MF, Workowski KA, Ganesh G, Jacob JT (2010). Aspergillus endocarditis: a review of the literature. Int J Infect Dis.

[2] Tattevin P, Revest M, Lefort A, Michelet C, Lortholary O (2014). Fungal endocarditis: current challenges. Int J Antimicrob Agents.

[3] Lim ML, Oliver DH, Barasch E (1997). Aspergillus mural vegetation identified by transesophageal echocardiography. Echocardiography.

[4] Alvarez JR, Quiroga JS, Taboada CR, Gonzalez AF, Torres FM, Garcia-Bengochea J (2004). Cardiac Aspergillosis with a Pedunculated Mass in the Left Ventricle. Tex Heart Inst J.

[5] Mullen P, Jude C, Borkon M, Porterfield J, Walsh TJ (1986). Aspergillus mural endocarditis. Clinical and echocardiographic diagnosis. CHEST J.

[6] Bajaj N, Chadha D, Hasija P, Arora HS. Invasive aspergillosis presenting as an Intracardiac mass in an immunocompetent host. BMJ Case Rep. 2016. 10.1136/bcr-2015-213205 PMC4785413 26944369

[7] Rajbanshi BG, Hughes JE, Desimone DC, Maleszewski JJ, Baddour LM, Dearani JA (2012). Surgical excision of invasive aspergillosis of the right ventricle presenting as intractable ventricular arrhythmia and right ventricular mass. Mayo Clin Proc.

[8] Walsh TJ, Anaissie EJ, Denning DW (2008). Treatment of aspergillosis: clinical practice guidelines of the Infectious Diseases Society of America. Clin Infect Dis.

